# Analysis of alternative signaling pathways of endoderm induction of human embryonic stem cells identifies context specific differences

**DOI:** 10.1186/1752-0509-6-154

**Published:** 2012-12-15

**Authors:** Shibin Mathew, Maria Jaramillo, Xinan Zhang, Li Ang Zhang, Alejandro Soto-Gutiérrez, Ipsita Banerjee

**Affiliations:** 1 Department of Chemical and Petroleum Engineering, University of Pittsburgh, 1249 Benedum Hall, 3700 O’Hara Street, Pittsburgh, PA 15261, USA; 2 Department of Bioengineering, University of Pittsburgh, 360B CNBIO, 300 Technology Drive, Pittsburgh, PA 15219, USA; 3 School of Mathematics and Statistics, Central China Normal University, Wuhan, China; 4 Department of Pathology, University of Pittsburgh, Pittsburgh, PA, USA; 5 McGowan Institute for Regenerative Medicine, University of Pittsburgh, Pittsburgh, PA, USA; 6 Thomas E. Starzl Transplantation Institute, University of Pittsburgh, Pittsburgh, PA, USA; 7 Center for Innovative Regenerative Therapies, Department of Surgery, Transplantation Section of Children's Hospital of Pittsburgh, Pittsburgh, PA, USA

**Keywords:** Human embryonic stem cells, Endoderm, Hierarchical clustering, Biclustering, Bootstrap

## Abstract

**Background:**

Lineage specific differentiation of human embryonic stem cells (hESCs) is largely mediated by specific growth factors and extracellular matrix molecules. Growth factors initiate a cascade of signals which control gene transcription and cell fate specification. There is a lot of interest in inducing hESCs to an endoderm fate which serves as a pathway towards more functional cell types like the pancreatic cells. Research over the past decade has established several robust pathways for deriving endoderm from hESCs, with the capability of further maturation. However, in our experience, the functional maturity of these endoderm derivatives, specifically to pancreatic lineage, largely depends on specific pathway of endoderm induction. Hence it will be of interest to understand the underlying mechanism mediating such induction and how it is translated to further maturation. In this work we analyze the regulatory interactions mediating different pathways of endoderm induction by identifying co-regulated transcription factors.

**Results:**

hESCs were induced towards endoderm using activin A and 4 different growth factors (FGF2 (F), BMP4 (B), PI3KI (P), and WNT3A (W)) and their combinations thereof, resulting in **15** total experimental conditions. At the end of differentiation each condition was analyzed by qRT-PCR for **12** relevant endoderm related transcription factors (TFs). As a first approach, we used hierarchical clustering to identify which growth factor combinations favor up-regulation of different genes. In the next step we identified sets of co-regulated transcription factors using a biclustering algorithm. The high variability of experimental data was addressed by integrating the biclustering formulation with bootstrap re-sampling to identify robust networks of co-regulated transcription factors. Our results show that the transition from early to late endoderm is favored by FGF2 as well as WNT3A treatments under high activin. However, induction of late endoderm markers is relatively favored by WNT3A under high activin.

**Conclusions:**

Use of FGF2, WNT3A or PI3K inhibition with high activin A may serve well in definitive endoderm induction followed by WNT3A specific signaling to direct the definitive endoderm into late endodermal lineages. Other combinations, though still feasible for endoderm induction, appear less promising for pancreatic endoderm specification in our experiments.

## Background

Embryonic stem cells have been shown to have tremendous impact in the field of regenerative medicine because of its potential to differentiate to multiple cell types of interest. Efficient harvesting of this potential requires careful development of protocols to evolve the cells through specific signaling pathways which will induce desired lineages and properties in the differentiated phenotypes. Our primary interest lies in differentiation of human embryonic stem cells (hESCs) to insulin producing β-cells of the pancreas as a cellular transplantation strategy for diabetes mellitus. The first and perhaps the most important step in differentiation to endodermal organs like pancreas and liver is the commitment to definitive endoderm (DE) [1]. Multiple signaling pathways have been reported to have success in inducing endoderm differentiation with subsequent maturation to liver, pancreas and lung. While there is some understanding of the activity pathway of these individual signaling molecules, detailed knowledge of transcriptional controls activated through these signaling pathways is largely unknown. Moreover, cooperative effect of these endoderm induction pathways, along with its impact on long term maturation has received less attention. Although standard protocols have been established for the later stages of pancreatic induction, it is not always obvious how these endoderm derivatives derived from different pathways will respond to subsequent pancreatic induction signals. In this article, we have analyzed the endoderm induction stage of the differentiation process induced by the combinatorial action of the signaling pathways using an integrated experimental and mathematical approach. A detailed mathematical analysis is adopted to capture co-regulated TFs across different growth factor combinations and projection of maturation potential of the various endoderm derivatives.

### Differentiation of hESCs to DE

Activin A (henceforth denoted as activin) has been shown to be effective in inducing DE from hESCs and is a key induction factor used in many protocols [2,3]. However, recent studies have shown that activin alone may not produce homogeneous differentiation and additional factors must be used to modulate supplementary signaling pathways along with the nodal pathway activated by activin [1,4]. We chose several widely used DE induction protocols all of which involve activin with either PI3K inhibition [5], WNT3A [3], BMP4 [6] or FGF2 [7]. The hESCs were differentiated into DE using these molecules alone and in all possible combinations, at the end of which the differentiated cell population was analyzed for endoderm markers. Our aim is twofold: to identify which growth factor combinations are most effective for efficient DE induction; and to understand TF interactions governing these induction conditions. We analyzed the mean expression data using Hierarchical clustering (HC) to identify relationships between the conditions and the TFs and biclustering on the original expression data with replicates to identify the TFs which are co-regulated under subsets of these conditions.

### Hierarchical clustering

HC is a useful technique to analyze and interpret multivariate data. Each data point here is represented as a vector and the distances between these data points are measured using a suitable distance measure [8]. The clustering process then links the data points together and the result is a hierarchical grouping of the data points in each of the dimensions (TFs and conditions in our case). Our primary goal in using HC is to capture the similarities between different growth factor treatments for DE induction as well as to identify co-regulated TFs under each of these treatments. HC has been successfully used in a number of bioinformatics applications including microarray data analysis, structure identification of bio-molecules and gene pathway identification [9].

### Biclustering to identify co-regulated genes across different conditions

While HC homogenizes the entire dataset, techniques like biclustering are useful in preserving the second dimension in clustering; in our case all the endoderm induction conditions. We are interested in identifying specific sets of genes exhibiting similar expression patterns across various subsets of experimental conditions, which can be achieved by biclustering. Likewise, many TFs are known to have multiple functions, and hence participate in multiple regulatory networks, which can also be captured by overlapped biclusters [10]. In 2000, Cheng and Church proposed the use of a similarity measure called the mean square residue for identification of coherent biclusters [11]. Since then newer and better algorithms have been developed to identify biclusters with particular characteristic trends like coherence, low overlaps and hierarchical structure [12]. These algorithms perform either one or a combination of iterative row and column clustering, greedy iterative search, exhaustive bicluster enumeration or distribution parameter identification [13]. Bleuler *et al*. proposed an evolutionary algorithm (EA) to determine high quality, partially overlapped biclusters using the Cheng and Church formulation [14]. EAs have the advantage of large search space and are efficient methods for complex optimization problems [15]. High quality biclusters should satisfy many criteria; namely they should contain as many genes and conditions as possible, low mean square residue, high row variance and should have low overlapping. Divina *et al*. formulated Sequential Evolutionary Biclustering (SEBI) algorithm to identify such biclusters from the expression data which has been adopted in the current work to identify important biclusters for the endoderm induction data under different combinations of the growth factors [15]. SEBI can find high quality biclusters and has been proved to perform well for large-scale biological datasets [15]. At the same time, it allows the user the flexibility of selecting the degree of overlap of the biclusters.

### Handling data variability

The gene expression data obtained for cell culture systems are subjected to noise because of the heterogeneity and stochasticity associated with the system. Differences among the biological replicates may therefore arise due to the inherent heterogeneity of the ES cell population as well as by experimental noise [16]. Therefore, it is essential that the biclustering algorithm be supplemented with additional methods to discover good quality and robust biclusters from noisy gene expression data. One way to do this is to obtain a large number of experimental replicates and perform biclustering over the entire dataset. This is however, expensive and impractical. A mathematical surrogate of this approach is bootstrapping, a concept first presented systematically by Efron *et al*. [17].

Essentially, bootstrapping generates a pseudo dataset from the small number of experimental replicates by a sampling with replacement technique. The advantage of bootstrap lies in estimating statistically significant parameters from a limited number of experimental replicates [18]. Thus, the results from a bootstrap analysis can provide information on the parameter variances and confidence intervals. These bootstrap data-sets are further analyzed by ensemble methods like bagging to identify aggregation of biclusters, referred to as meta-clusters [19]. We have adopted a similar approach to aggregate the individual biclusters identified from the bootstrap datasets. However instead of identifying an ensemble of biclusters, we have concentrated on identifying the most repeated subset of the bicluster, which we denote as robust.

## Results

**Figure 1:**
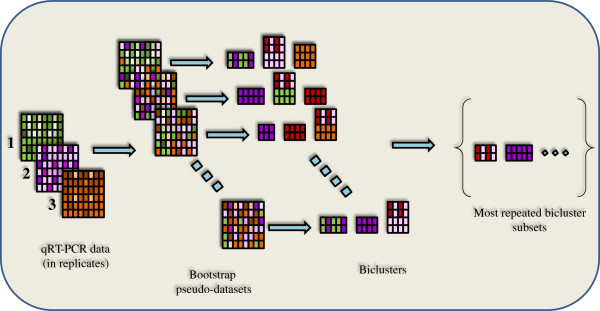
**Work-flow for the entire analysis from data collection to identification of robust biclusters**. In short, we start with the qRT-PCR data and perform bootstrap with re-sampling to obtain 1000 pseudo-datasets. Each of these datasets is subjected to biclustering analysis to obtain the most coherent pattern in each dataset. The resulting biclusters are then analyzed for the most repeated subsets of biclusters.

The focus of this work is to understand the mechanism of endoderm induction using different growth factors, acting alone and in combination, from an integrated experimental and computational approach (summarized in Figure 1). The H1 human embryonic stem cells were induced towards endoderm lineage using activin along with alternate growth factors, namely FGF2, BMP4, PI3KI, WNT3A, added in 15 combinations. The cells differentiated thereof were analyzed in detail for their gene expression levels, specifically concentrating on a broad range of endoderm markers along with representative pancreatic endoderm markers.

### Experimental analysis of endoderm differentiation using combinations of major pathways

**Figure 2:**
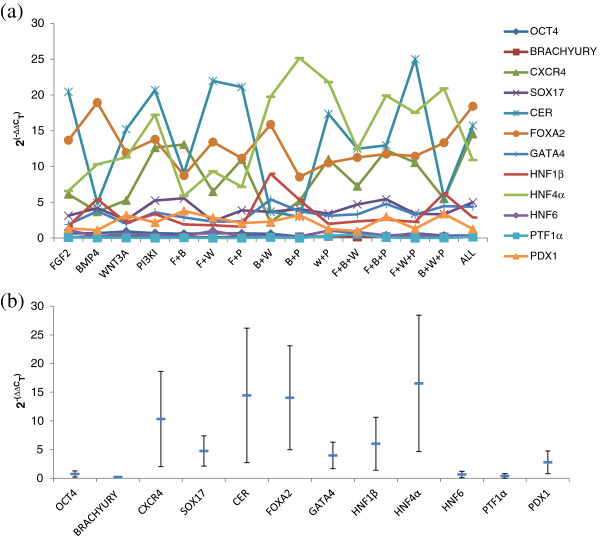
**Fold change data for the 12 transcriptional markers across 15 experimental conditions.** (**a**) The fold change calculated from the mean expression data from qRT-PCR on day 4 of the differentiation process is plotted from the expression matrix, *X*, constructed using rows as the TFs and columns as the experimental conditions. (**b**) Variation observed in the 12 transcriptional markers with changes in the signaling pathways presented as mean ± SE. All the major DE markers *CER*, *CXCR4*, *FOXA2*, *SOX17* and the later endoderm markers *HNF4α*, *HNF1β* and *GATA4* show significant changes with the nature of DE induction.

Figure 2a shows the mean expression data plotted as fold changes in 12 genes across the 15 experimental conditions. At this stage, the fold change data showed interesting trends for the different conditions. When using only one factor other than activin, PI3KI along with activin was found to give the highest expression of most of the DE markers while BMP4 and activin in combination was found to give the lowest expression among the four conditions. Interestingly, BMP4 was found to perform better in combination with another factor like WNT3A or FGF2. Also, FGF2 containing conditions were found to favor *CER* while BMP4 containing conditions to favor *HNF4α*. Among the 4 conditions which contain 3 factors other than activin, combinations of FGF2, BMP4 and PI3KI perform well. Using all the factors together was not particularly useful since all the TFs maintained expressions in the same range as other combinations. Figure 2b shows the range of variation observed in each of the transcriptional markers across the 15 experimental conditions along-with the experimental replicates. The levels of DE markers *CER*, *FOXA2*, *CXCR4* and late endoderm markers *HNF4α*, *HNF1β* and *GATA4* change substantially when the induction conditions are changed. This level of analysis, however, makes it difficult to draw mechanistic insights from the dataset. Hence, we performed a more rigorous mathematical analysis to separate out the TF trends and associate them with the appropriate conditions. Because of the inherent differences in expression level of different genes, it is essential to normalize the data to avoid bias. For the mathematical analysis, the data presented in Figure 2a was normalized by mean centering and variance scaling so that every TF has a mean expression value of zero and standard deviation of one.

### Hierarchical clustering of the mean expression data identifies differences in the endoderm induced by BMP4 in the presence and absence of exogenous FGF2

**Figure 3:**
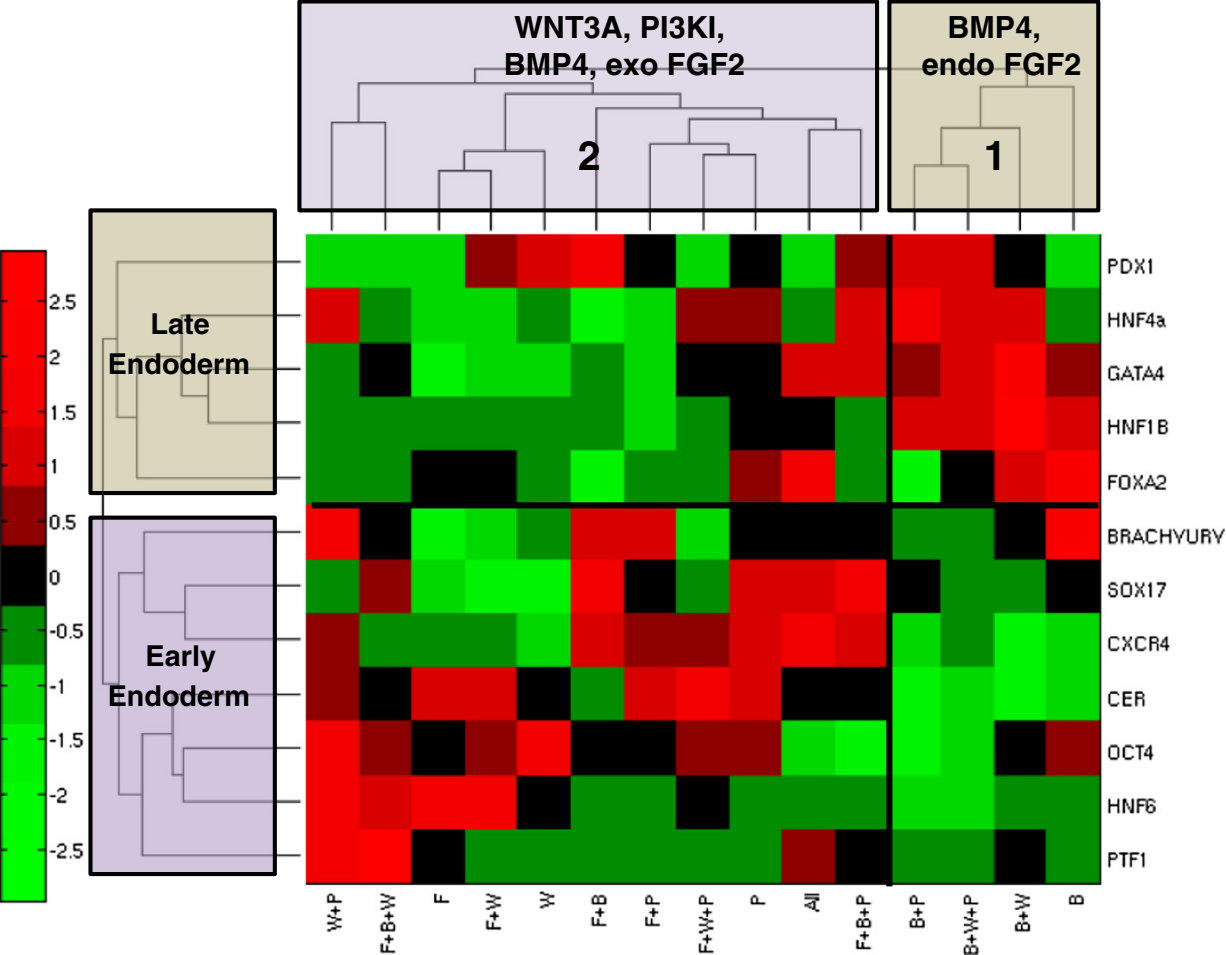
**Hierarchical clustering on the mean expression data.** The conditions cluster into two major groups, one containing BMP4 in the absence of exogenous FGF2 and the other containing all the other treatments and BMP4 in combination with exogenous FGF2. Activin A is common among all the treatments. The TFs cluster into two groups, the late and early endoderm markers.

The mean experimental data matrix was first analyzed using hierarchical clustering which clusters the TFs and conditions separately, as shown in Figure 3. Among the conditions, two major branches were observed: the first cluster contains BMP4 dominant conditions (B, B + W, B + P, B + W + P) and the second cluster contains the remaining conditions which also includes BMP4 but interestingly only in combination with FGF2. The TFs also segregate into two branches; the first branch contains the late endoderm markers and one of the DE markers (*HNF4α*, *HNF1β*, *GATA4*, *PDX1*, *FOXA2*), the second branch contains the early DE and late endoderm markers (*OCT4, BRACHYURY*, *CER*, *HNF6*, *CXCR4*, *SOX17*, *PTF1α*). The first group of markers is particularly high in BMP4 dominant conditions and low in the other conditions. The second group of markers is low in the BMP4 dominant conditions and high in the presence of PI3KI, WNT3A and BMP4 and high FGF2. Thus our results point to differences in activin and BMP4 induced endoderm in the presence and absence of exogenous FGF2. We performed principal component analysis (PCA) on the same data retaining only the first three components to filter noise and identify the most represented groups. As shown in the Additional file 1, a similar conclusion can be drawn from PCA further supporting our analysis.

The clusters identified by the hierarchical algorithm reflect our biological understanding of the induction conditions as seen from the previous studies. A major difference between the two clusters of conditions was the context dependent function of BMP4. In the presence of FGF2 and high activin, BMP4 was found to favor the endodermal lineage which was seen in several recent studies [20-22] and was also on par with PI3KI dominant conditions which gave the best endoderm in our experiments. Also, in our BMP4 dominant conditions, the late stage markers showed very high expression while the major DE markers were low indicating that the resulting endoderm may already be mature. Among the second group of conditions, PI3KI and high activin resulted in high expression of three major DE markers *SOX17*, *CXCR4* and *CER* which is supported by a number of earlier studies [23,24]. Using all the factors together does not improve upon the endoderm derived by PI3KI treatment. The second group of conditions also contains FGF2 as a major factor along with WNT3A. It is found that both pluripotency (*OCT4*) and the endoderm factors (*CER* and *HNF6*) are relatively favored by conditions involving FGF2 and WNT3A as the major contributor. In fact, FGF2 has been found to be sufficient to maintain the hESCs in the pluripotent state [25] and has also been used for endoderm induction in several differentiation protocols [26]. Thus, FGF2 can potentially favor both pluripotency as well as endoderm differentiation depending on associated conditions.

### Identification of co-regulated transcription factors by biclustering

While hierarchical clustering enables a fast and simplistic analysis of the experimental data sets, it does not provide information on which subsets of TFs are co-regulated across subsets of conditions. Identifying such co-clusters will be beneficial, since the governing signaling pathways change with the induction condition and the same TFs may not be co-regulated. The technique of biclustering serves to mine subgroups of such TFs exhibiting similar trends in their expression level under subsets of conditions. Hence TFs appearing in the same bicluster can be inferred to be co-regulated and constituents of a similar network architecture. The experimental data matrix, *X*, constituting the mean expression data across all the growth factor conditions is analyzed using the algorithm elaborated in Methods section. Here, the biclustering approach is formulated as an optimization problem solved using genetic algorithm (GA) and the quality of every candidate bicluster is assessed by a fitness function. The fitness function has a number of free parameters associated with it which can be tuned in order to identify certain desired trends. The detailed procedure on the selection of the optimum parameters is outlined in the Additional file 2.

**Figure 4:**
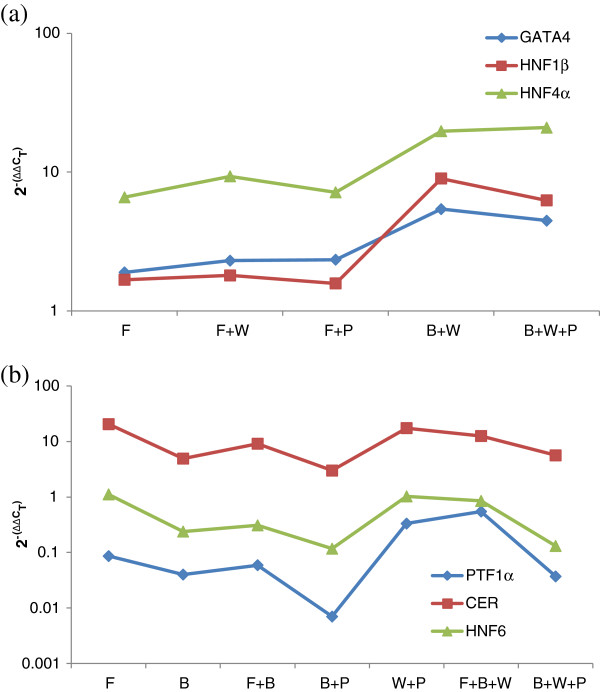
**Biclusters obtained from the normalized mean expression data.** (**a**) Optimal Bicluster The bicluster contains 3 genes across 5 conditions. (**b**) **Subsequent bicluster** containing 3 genes and 7 conditions. The bicluster parameters selected were *δ* = 1.5, *Wc*, *Wr* = 1.

The developed optimization based bicluster identification algorithm was applied to the mean expression data with the above mentioned parameters, which resulted in a 3-gene 5-condition bicluster as illustrated in Figure 4 (a). However, to identify additional biclusters, possibly with overlaps, the SEBI algorithm was subsequently run by penalizing the identified biclusters. One such bicluster is presented in Figure 4 (b). Although, the SEBI algorithm allows some degree of overlapping amongst the subsequent biclusters, the current mean dataset did not result in any overlaps.

Recently, a new method was proposed by Banka *et al*. called as Fuzzy Possibilistic Biclustering which assigns a membership value to each gene-condition pair in the expression matrix and therefore, allows varying degree of overlapping amongst the biclusters [27,28]. However, though the method has been proven to provide very large biclusters with acceptable residue, the selection of the degree of fuzziness often depends upon the question that the biologists have set to answer [29]. In our case, we are interested in analyzing the well identified markers of endoderm induction under necessary signaling pathways. Since, our aim is to discover subtle differences in the gene regulation when the induction conditions are changed, a traditional crisp method like SEBI will be more useful for identifying the best induction condition.

### Robust biclusters identify WNT3A treatment to favor both early and late endoderm

The above identified biclusters were for the mean dataset, and hence does not explicitly take into account the experimental variations. In general biological datasets are known for their noise and uncertainty, and in particular stem cells have inherent heterogeneity and stochasticity. In order to increase confidence in the identified bicluster we undertook bootstrap analysis on the experimental data to generate 1000 pseudo-datasets. Each of these datasets were treated as an experimental repeat and subjected to the entire biclustering analysis. In order to identify somewhat overlapped biclusters, we ran the biclustering algorithm five times at each data point by subsequently penalizing previously identified biclusters.

**Figure 5:**
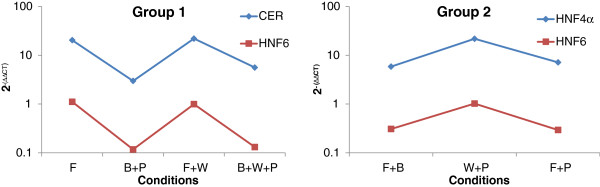
**Robust subsets identified from the 1000 bootstrap datasets.** Robust biclusters are the most repeated subsets (>500). The bicluster parameters selected were *δ* = 1.5, *Wc*, *Wr* = 1. Note: Group 1 contains five subsets only one of which is shown.

The next task was to determine a robust bicluster from this array of alternate biclusters. We hypothesize that the robust bicluster will not be significantly affected by the experimental noise, and hence will appear a large number of times in the bootstrapped-bicluster data set. However, a thorough search of the entire array of alternate biclusters for frequency of repeats did not yield any satisfactory outcome. Thus we could not find a single bicluster that was significantly repeated in its entirety across the data set. Instead, we realized subsets of genes and conditions of the bicluster were being repeated with very high frequency instead of the entire bicluster. Hence, we focused on identifying such subsets from the family of bootstrap + bicluster solutions. Setting a minimum threshold of 50% repeats across the bootstrap samples, we identified 6 such subsets. First five of these contained different combinations of the same two markers and four conditions. Hence we collected them together into a single group. The profiles of the repeated subsets are presented in Figure 5. These subsets are of two kinds: Group 1 contains (*CER*, *HNF6* | F, F + W, B + W + P, B + P) and Group 2 contains (*HNF6*, *HNF4α* | F + B, F + P, W + P). It is important to note that the robust biclusters were different from the biclusters obtained for the mean expression data. For example, the biclusters in Figure 4 show that *HNF4α* clusters closer to *HNF1β* (and *GATA4*) rather than *CER*. This is also evident from our hierarchical clusters in Figure 3. The fact that they do not appear together in the robust biclusters is interesting and shows that analysis from mean datasets can be risky for stem cell systems when there is inherent variability among the replicates. Supportively; the *HNF4α, HNF1β* (and *GATA4*) combination occurs in subsets with less than 300 repeats (data not shown).

**Figure 6:**
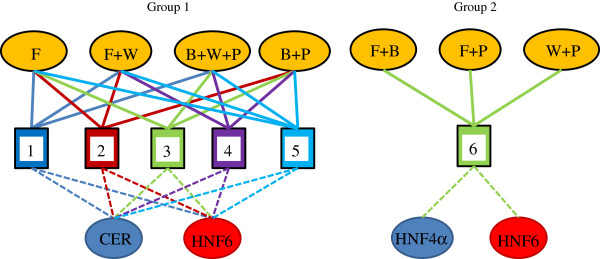
**Robust subsets of co-regulated genes presented as a bipartite graph.**. We have identified high Activin along with PI3K inhibition or activin in combination with WNT3A to work the best to co-regulate early endoderm marker *CER* and late endoderm markers *HNF6*. The Group 2 TFs *HNF4α* and *HNF6* are part of the network inducing *NGN3* and *PDX1*, reminiscent of the pancreatic genotype and are favored by high activin with PI3KI and WNT3A.

Figure 6 shows a summary of the robust biclusters represented as a bipartite graph of genes and conditions. The identified biclusters are biologically relevant to the development stages *in vivo*. Group 1 contains endoderm markers *CER* and *HNF6* under FGF2/WNT3A and BMP4/WNT3A/PI3KI. *CER* is an important early marker for the DE stage rising after the formation of the primitive streak during development while *HNF6* is a marker for a more primitive foregut stage in pancreas development [2]. Thus, Group 1 is similar to the foregut development stage *in vivo*[30]. In addition, the conditions in Group 1 contain FGF2 and WNT3A but not BMP4 and as seen from Figure 5, *CER* and *HNF6* decrease under BMP4 dominance. Thus, the biclustering analysis shows that the early marker *CER* and a late endoderm marker *HNF6* are controlled by the FGF2, WNT3A pathway and are relatively down-regulated under BMP4 and PI3KI. Group 2 contains another primitive foregut stage marker *HNF4α* along-with *HNF6*[2]. Interestingly here, the biclustering results show that pancreatic endodermal transcriptional machinery may not be favored at the DE stage by the FGF2 + BMP4 combination although in our hierarchical clustering results FGF2 + BMP4 combination clustered with the other conditions that gave a better DE signature. We also note that WNT3A and PI3KI combination with high activin increased the expression of *HNF4α* and *HNF6* and these conditions also gave a successful DE signature as seen from the hierarchical clustering. Thus our results indicate that WNT3A pathway can favor both early and late markers like *CER*, *HNF4α* and *HNF6.* Also, WNT3A + PI3KI induced DE cells may be more capable of developing into later pancreatic lineages. While WNT3A and PI3KI have been used for DE induction towards pancreatic maturation [3,5], the effect of co-induction has not been explored yet.

## Discussion

The differentiation of hESCs into the endoderm lineages is carried out by the activation of different signaling pathways mimicking *in vivo* development. However, there is no consensus on which induction method is the most desirable and whether combination of these could result in an endoderm with the best signature. Here, we have used a combination of experimental and mathematical techniques to shed light on these concerns.

### The DE signature differs under exogenous activation of different signaling pathways participating in endoderm commitment

Our experiments with different DE inducing conditions show that the DE potential of the differentiating hESCs is highly dependent on the method of DE induction. The major DE markers (*CER*, *CXCR4*, *FOXA2*, *SOX17)* showed considerable variation when some of the pathways were activated above their basal levels.

All the pathways studied here have been known to be important at the earlier stages of *in vivo* endoderm differentiation and has also been documented as necessary for *in vitro* differentiation [2,6,7,31]. The common denominator in our studies is activin A which is an essential inducer of DE [2,3,24]. This is primarily because activin, being a member of the TGFβ family, mimics nodal signaling which is proven to be necessary for endoderm development [4]. Activin has been shown to maintain pluripotency at low concentrations and to induce mesoderm and endoderm at high concentrations [25]. However, activin alone may not result in efficient endoderm induction [1]. Low PI3K signaling was essential for efficient induction of DE from hESCs [24]. Our hierarchical clusters show that Activin and PI3K inhibition in combination favor the up-regulation of a number of DE markers and form the most minimal signaling pathways to be modulated for efficient DE induction. In fact a number of recent studies have identified the interplay between PI3K/Akt and Activin/Smad2,3 pathways and the resulting regulation of the gene transcription events necessary for early DE induction [23].

Among the DE markers, *CER* showed up-regulation on differentiation, and the highest up-regulation was achieved in the presence of FGF2, WNT and PI3KI treatments. Katoh *et al*. recently identified the binding domains of several key signaling effectors of the activin and WNT pathways on the promoter regions of *CER* in hESCs [32]. According to their results, the key nodal effectors Smad3/Smad4 as well as the WNT effectors beta-catenin and TCF/LEF transcriptional complex regulate the expression of the *CER* gene. In addition to high activin and WNT signaling, PI3K inhibition may be necessary to enhance the effect of nodal signaling as Smad3/Smad4 complex is negatively regulated by Akt [23]. Exogenous FGF2 simultaneously activates the ERK pathway and maintains the expression of other key regulators of differentiation [33]. However, BMP4 effectors Smad1/3 may compete with the activin pathway and thus reduce the up-regulation of *CER*, as substantiated by the consistent grouping of the BMP4 dominant conditions in the hierarchical clustering with low *CER* as a common marker.

The response to the BMP4 pathway, however, was highly dependent on the context, namely the presence and absence of FGF2 which was a striking feature of the hierarchical clustering on the 15 conditions. BMP4 is typically known as an activin antagonist and high concentrations of BMP4 in the culture with high activin results in mesoderm fate [34-36]. At the same time, BMP4 alone results in the extra-embryonic lineages [37]. The presence of FGF2 with BMP4 modulates the net response to the mesendoderm fate, which is an intermediate stage that can result in DE and mesoderm. Several recent studies have demonstrated the use of this combination to promote endoderm formation [21,22,38]. FGF2 sustains the expression of Nanog (a pluripotency marker) and this sustained Nanog expression is found to shift the outcome of BMP4 induced differentiation of hESCs towards mesendoderm [22]. However, prolonged use of FGF2 and BMP4 together may be detrimental for pancreatic differentiation, since this combination has been shown to induce hepatic differentiation after the DE stage [30]. Also, BMP4 dominant clusters showed high expression of late endoderm markers *HNF4α*, *HNF1β* and *GATA4*. This may indicate that BMP4 accelerates the differentiation to the mesendoderm phase and therefore, the overall dynamics may be faster for the BMP4 dominant case. But, it was striking to note that the expression of *HNF6*, another important marker for late endoderm was still lower in the BMP4 dominant case. Hence, hierarchical clustering alone was not sufficient to answer if BMP4 addition could be useful for late endoderm differentiation. Importantly, BMP4 dominant conditions gave low expression of markers from the robust biclusters. Thus the current analysis shows that BMP4 may not be a suitable choice for endoderm induction.

WNT3A/β-catenin signaling has been shown to be important both for maintenance of pluripotency as well as induction of differentiation [25]. The WNT pathway is also found to be important in the formation of primitive streak due to which it is often used in the very early stages of *in vitro* differentiation until the formation of mesendoderm [2]. Stabilization of β-catenin by canonical WNT signaling is found to be responsible for differentiation by epithelial-mesenchymal transition;, however presence of Wnt after this stage supports mesoderm [36]. Also, FGF2 is found to synergistically influence the WNT pathway [39]. WNT alongwith PI3KI was commonly present in both the groups identified by our hierarchical clustering. WNT was consistently found to be supportive to the activin + FGF2 signaling assessed by the up-regulation of DE markers. Hence, WNT and PI3KI may be the essential pathway modulators necessary for endoderm differentiation.

### Robust biclusters identify the necessary pathways for efficient endoderm differentiation to the pancreatic lineage

The robust biclusters identified by the biclustering + bootstrap analysis show the most important trends preserved under experimental variations. Supportively, *CER, HNF6* and *HNF4α* belonged to the robust clusters. As mentioned earlier, *CER* is an important target of the activin and WNT signaling pathways and *HNF6* is a very early pancreatic progenitor marker taking part in the transcriptional network activating pancreatic progenitors [32,40]. As seen from the Group 1 bicluster, FGF2 + WNT3A conditions favor *CER* and *HNF6* while BMP4 limits their up-regulation. It is also found that the stability of β–catenin is partly enhanced by PI3K signaling (activated by FGF2) [41] and hence this combination of high activin + FGF2 + WNT3A may work to control the expression of some endoderm markers like *CER* and *HNF6*. At the same time, CER protein is a negative regulator of the Tgfb (activin, BMP4) pathway and up-regulation of *CER* is necessary to limit the activation of these pathways, since inhibition of the Tgfb pathway was found to be necessary for efficient differentiation to the pancreatic progenitors after *PDX1* and *HNF6* expression [42]. However, external addition of WNT3A may still be necessary since *CER* negatively regulates the WNT pathway [32].

**Figure 7:**
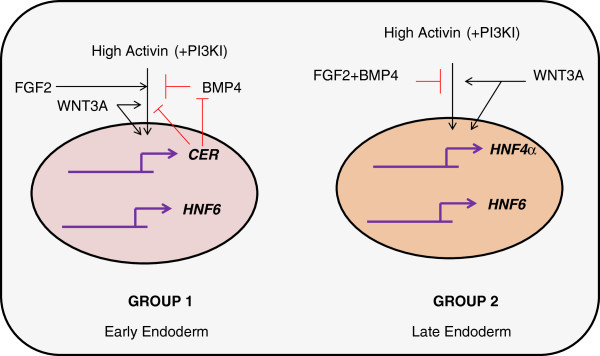
**Figure summarizing the functional dependence of the co-regulated genes on the active signaling pathways of endoderm induction. ***CER* and *HNF6* are favoured by High activin and PI3KI, WNT3A, FGF2 while *HNF4α* and *HNF6* are favoured by High activin, WNT3A and PI3KI. Combining the early and late stages, high activin with PI3KI and WNT3A together is an effective strategy for endoderm differentiation.

Alternatively, the markers *HNF4α* and *HNF6* which occur in Group 2 are co-regulated under FGF2 + BMP4, FGF2 + WNT3A + PI3KI action. These markers also occur in the MODY network for induction of Neurogenin expressing cells which represents mature pancreatic lineage [40]. *HNF6* occupies a predominant position in regulating the expression of *HNF4α* and other genes prior to *PDX1* induction. A key result identified by the bicluster was the consistent up-regulation of the late pancreatic markers *HNF4α* and *HNF6* under WNT3A + PI3KI dominant conditions and studies by Nostro *et al*. have indicated the necessity of WNT3A for induction of pancreatic progenitors [42]. *CER*, *HNF6* combination was also up-regulated under WNT3A conditions and thus WNT3A addition was found to favor both DE markers as well as late pancreatic endoderm markers supposedly showing similarity with *in vivo* pancreatic organogenesis. The presence of FGF2 and BMP4 lowers the expression of these markers and is consistent with the inhibition of FGF2 and BMP4 at the later stages for inhibition of a hepatic fate and efficient pancreatic lineage selection [42]. The key signaling pathway interactions from the robust biclusters are summarized in Figure 7.

## Conclusion

The focus of the current work was to achieve insights into the *in vitro* differentiation process of human embryonic stem cells to the endoderm stage using both experimental and mathematical approaches. Our work has identified the differences between the different protocols for endoderm induction. Essentially, high activin A and PI3K inhibition or high activin A with FGF2 or WNT3A serve well as early DE inducer. Additionally, biclustering shows that the early and late endoderm markers are co-regulated under high activin and WNT3A. Thus, overall high activin with PI3KI and WNT3A together may serve better for *in vitro* differentiation of hESCs to the definitive endoderm and pancreatic endoderm lineages.

## Methods

### Experimental methods

#### Cell culture and treatment

##### hESC maintenance

H1 hESCs were placed on hESC certified matrigel coated wells and maintained with mTeSR1 with media change every day. Cells were passaged every 5 to 7 days by incubating in 1 mg/ml dispase for 5 minutes followed by mechanically breaking the colonies and splitting at a 1:3–1:5 dilution. Cells were examined under the microscope every day and colonies with observable differentiation were picked and removed before the media changes.

##### hESC differentiation to DE

H1 hESCs were allowed to grow to 60-70% confluency before the experiments were started. Once confluency was reached, differentiation was performed by adding DE induction media for 4 days with media change every day. Several induction conditions were chosen according to previously published studies [3,5-7]. All conditions were prepared in DMEM:F12 supplemented with B27 and 0.2% BSA with 100 ng/ml Activin A. Conditions involved the use of individual and all possible combinations of growth factors and molecules at the following concentrations: basic FGF (F) at 100 ng/ml, BMP4 (B) at 100 ng/ml, WNT3A (W) at 25 ng/ml and Wortmannin (PI3K inhibitor, P) at 1 μM. This leads to 15 different experimental conditions.

#### Measurement of Transcription Factor (TF) expression

After 4 days of DE induction, cells were lysed and RNA extracted using Nucleospin RNA II kit (Macherey Nagel) according to the manufacturer’s instructions. The sample absorbance at 280 nm and 260 nm was measured using a BioRad Smart Spec spectrophotometer to obtain RNA concentration and quality. Reverse transcription was performed using ImProm II Promega reverse transcription kit following the manufacturer’s recommendation. qRT-PCR analysis was performed for endoderm and pancreatic markers using the primers listed in Additional file 3: Table S1.

A total of 12 transcription factors were studied which included pluripotency marker *OCT4*, mesendoderm marker *BRACHYURY*, DE markers namely, *CXCR4*, *SOX17*, *CER*, *FOXA2* and pancreatic progenitor markers *PTF1α*, *PDX1*, *GATA4*, *HNF1β*, *HNF4α* and *HNF6*. *GAPDH* was selected as the housekeeping gene. Briefly, the fold change was calculated from the cycle times, *C*_*T*_, after normalization with respect to the control sample and housekeeping gene, *GAPDH* as: $2^{-\Delta \Delta C_{T}}$, where, *ΔΔC*_*T*_ = [(*C*_*T*,*target*_ − *C*_*T*,*GAPDH*_)_*sample*_ − (*C*_*T*,*target*_ − *C*_*T*,*GAPDH*_)_*undiff cells*_]. The control sample was chosen to be undifferentiated cells at day 0.

#### TF expression profiles

The TF expression profiles can be grouped together to form an expression matrix with the rows corresponding to the measurements of interest (like the relative mRNA concentrations) and the columns corresponding to the experimental conditions or samples. Thus, each element in the matrix refers to the intensity of the particular measurement in a given sample [43]. Many of the genes are closely regulated under a subset of conditions indicating that they are probably under the influence of the same regulatory network under these conditions [12]. The expression data is helpful in identifying such sub groups of transcription factors and conditions. However, expression data matrices are often complex and further computational analysis is required to mine important connections from such large expression matrices.

### Mathematical analysis

#### Hierarchical clustering

Hierarchical clustering partitions the data into clusters through an iterative process, where similarity or dissimilarity between every pair of variables in the data matrix is calculated using an appropriate distance measure followed by grouping the variables in close proximity using a linkage function. We used the in-built Matlab functions to perform the analysis using various distance measures e.g. Euclidean, city block etc., on the mean centered and variance scaled expression matrix. The results were represented as a clustergram i.e. the linkage tree and the corresponding heat map. We tested the tree generated using different linkage measures after normalization of the mean expression matrix and found all the trees to be very similar with the cophenetic correlation coefficient greater than 0.9.

#### Biclustering algorithm

Biclustering can be described as two dimensional clustering, where a subset of genes exhibiting similar trend across a subset of conditions is being identified. Such subsets can be considered to be participating in similar regulatory mechanism, hence constituting a regulatory network. In order to identify sets of TFs expressing coherent trends under specific sets of conditions, we analyzed our TF-condition matrix, *X*, using the Sequential Evolutionary Biclustering (SEBI) developed by Divina *et al*. [15]. The SEBI algorithm identifies coherent biclusters sequentially with the help of a number of metrics as described below. For a bicluster *B*(*I*, *J*) ∈ *X*, containing elements, *e*_*ij*_ for *i* ∈ *I*, *j* ∈ *J*, the residue, *r*_*ij*_ of each element in the bicluster is defined as: *r*_*ij*_ = *e*_*ij*_ − *e*_*iJ*_ − *e*_*Ij*_ − *e*_*IJ*_. The gene base is defined as $e_{\mathrm{iJ}}=\frac{\sum_{j\in J}e_{\mathrm{ij}}}{\left|J\right|}$, with *I* and *J* representing the total number of genes and conditions respectively in the bicluster *B*. The condition base is defined as $e_{\mathrm{Ij}}=\frac{\sum_{i\in I}e_{\mathrm{ij}}}{\left|I\right|}$. The base of the bicluster is the mean of all entries in the bicluster, i.e., $e_{\mathrm{IJ}}=\frac{\sum_{i\in I,j\in J}e_{\mathrm{ij}}}{\left|I\right|\times \left|J\right|}$. The residue, therefore, indicates the degree of coherence of the element with other elements in the bicluster. Further, the squared mean residue of all the elements in the bicluster is defined as $r_{\mathrm{IJ}}=\frac{\sum_{i\in I,j\in J}{r^{2}}_{\mathrm{ij}}}{\left|I\right|\times \left|J\right|}$. It is possible to have biclusters having constant expression values and hence have low residue value. To avoid such trivial biclusters, the variance metric is introduced. The variance, var_*IJ*_, of a bicluster is defined as, ${\mathrm{var}}_{\mathrm{IJ}}=\frac{\sum_{i\in I,j\in J}{{\left(e_{\mathrm{ij}}-e_{\mathrm{iJ}}\right)}^{2}}_{\mathrm{ij}}}{\left|I\right|\times \left|J\right|}$. Hence, the variance captures fluctuating trends. Finally, we would be interested in biclusters with as many genes and conditions as possible i.e. having large volume. The basic premise of the analysis is that the genes belonging to a bicluster are under the influence of a common regulatory pathway and hence show coherence in their expression trends. However it is possible for the genes to participate in multiple regulatory pathways, to capture which we allow certain degree of overlapping amongst the biclusters discovered sequentially by the SEBI algorithm using a penalty term.

Thus, our final goal is to find biclusters of maximum size, with mean squared residue lower than a given threshold (*δ*), with relatively high row variance, and a low level of overlapping among the biclusters. We represent this as an optimization problem with objective function defined as [15]:

(1) $$\min_{X},F\left(B\right)=\frac{m\_\text{residue}\left(B\right)}{\delta }+\frac{1}{\mathrm{row}\_\text{var}\text{iance}\left(B\right)}+w_{d}+\mathrm{penalty}$$

In this function, *B* (*I*, *J*) is an individual solution, *m*_*residue* is the mean squared residue of the bicluster *B*, *row*_var*iance* (*B*) is the row variance of *B*, $\mathrm{penalty}=\sum_{i\in I,j\in J}w_{p}\left(e_{\mathrm{ij}}\right)$, where *w*_*p*_ is defined as

$$w_{p}\left(e_{\mathrm{ij}}\right)=\{\begin{array}{ll} 0 & if\left|\mathrm{Cov}\left(e_{\mathrm{ij}}\right)\right|=0\\ \frac{\sum_{n\in N,m\in M}\left|\mathrm{Cov}\left(e_{\mathrm{nm}}\right)\right|}{e^{-\left|\mathrm{Cov}\left(e_{\mathrm{ij}}\right)\right|}} & if\left|\mathrm{Cov}\left(e_{\mathrm{ij}}\right)\right|>0 \end{array}$$

Where *N*, *M* are the number of rows and columns of the expression matrix, respectively and |*Cov*(*e*_*ij*_)| is the number of previous biclusters containing *e*_*ij*_. The use of the penalty term biases the search against members which already have appeared in the previous biclusters, thus reducing the overlapping amongst the biclusters.

*w*_*d*_ is defined as $\left(w_{r}\cdot \frac{\delta }{\mathrm{ro}w_{B}}+w_{c}\cdot \frac{\delta }{\mathrm{colum}n_{B}}\right)$ and *δ* is the threshold mean squared residue and biclusters with mean squared residue above *δ* are discarded.

#### Solution procedure

The current optimization formulation has been identified to be NP-hard and has been shown to be effectively handled by evolutionary techniques like Genetic Algorithm (GA) [15]. GA is an iterative search process which looks for the fittest member of a population (candidate solutions) using the biological principle of evolution under mutation and natural selection [44]. In a typical GA, the optimization variables are encoded as a sequence of binary bits and these sequences are concatenated to form the chromosome. Thus, for the present formulation, each chromosome consists of *I* binary bits for genes and *J* binary bits for conditions forming the *I* + *J* binary bits of the chromosome. The binary variables, 0 and 1 represent the absence or presence of a gene (or condition) respectively. Thus, a GA population is made of chromosomes with each chromosome representing a candidate bicluster.

Each chromosome has a metric associated with it called the fitness which we wish to maximize. The GA algorithm is initiated by randomly initializing a population of chromosomes (i.e. biclusters). The population is continuously evolved in every generation by the operators: reproduction, crossover and mutation. At the end of every generation, individuals for the next one are selected on the basis of their fitness values. This cycle of evolution is continued until a predetermined termination criterion is reached. For the present case, we continued the simulations for a maximum number of generations until no further change in the population was observed. The biclustering formulation was coded in FORTRAN R90 and the Genetic Algorithm (version 1.7a) driver obtained from David Carroll, CU Aerospace, Urbana, IL. Computations were performed on INTEL (R) Core (TM) 2 Quad CPU (Q8400 @ 2.66 GHz).

#### Determination of robust biclusters

The inherent noise in biological systems makes it difficult to draw meaningful conclusions from a deterministic analysis. The formulation proposed above is based on the mean gene expression data which possibly reduces confidence in the identified bicluster. Here we have adopted the bootstrap technique to obtain robust biclusters from noisy experimental data. Bootstrap is a statistical technique to generate large data set from a small number of experimental replicates, using sampling with replacement technique. The present formulation systematically re-samples the original experimental data set using Monte Carlo algorithm to generate the artificial data set. The optimization formulation of the biclustering problem is then solved at each of the bootstrap data points to generate a family of alternate biclusters. The final goal will be to identify the most repeated biclusters in the entire array, based on the justification that such a bicluster will be relatively insensitive to experimental noise and hence is robust. To this end, the number of repeats of a particular gene-condition combination is analyzed using the quicksort algorithm (N log N). Our analysis showed that the complete bicluster was typically not repeated significantly; instead only subsets of the biclusters were repeated sufficient number of times. For identification of robust biclusters, we set the threshold frequency of repeats as 500 out of every 1000 alternate biclusters. The most repeated subsets are thereby concluded to be robust under experimental noise. The work flow for the entire analysis is depicted in Figure 1.

## Competing interests

The authors declare that they have no competing interests.

## Authors’ contributions

Conceived and designed experiments: IB MJ ASG SM. Performed the experiments: MJ. Conducted mathematical analysis: SM, XZ, LZ. Contributed materials/analysis tools: IB. Drafted the manuscript: SM IB. All authors read and approved the final manuscript.

## Supplementary Material

Additional file 1 Principal Component Analysis.docx Click here for file

Additional file 2 Selection of biclustering parameters.docx Click here for file

Additional file 3 Transcription factors and primers list.docx Click here for file
