# Supplementary material for: Analysis of alternative signaling pathways of endoderm induction of human embryonic stem cells identifies context specific differences
Source: BMC Syst Biol. 2012 Dec 15;6:154. doi: 10.1186/1752-0509-6-154 (PMC3547704; doi:10.1186/1752-0509-6-154)
Supplement: Additional file 1 — Principal Component Analysis.docx [file 1752-0509-6-154-S1.docx]

*Principal Component Analysis*

The expression level of all the 12 TFs are analyzed for each of 15 conditions (chosen from F, B, W, P and all combinations thereof), and expressed as fold change of expression level with respect to undifferentiated cells at day 0. The mean fold change data is tabulated as a 12 15 expression matrix ($X$) with 12 genes as the rows and 15 experimental conditions as columns. Prior to the analysis, the matrix, $X$, was normalized by mean centering and variance scaling across each TF. PCA was done on the mean expression data in MATLAB R2010 by using the *princomp* option. The method uses Singular Value Decomposition (SVD) to factorize the matrix into scores and loadings matrices respectively. The scores matrix, $F$, represents the observations (i.e. experimental conditions) in the principal component space while the loadings matrix, $Q$, represents the principal components (PCs) in terms of original variables (i.e. TFs) such that $X^{T}=FQ^{T}$. Thus, the PCA transforms the observations in terms of a new orthogonal coordinate space defined by the PCs. The first PC explains the largest variation in the data and the next PC captures the next largest and so on. Each of these PCs are related to the $I$ eigen values of the correlation matrix, $XX^{T}$, arranged in the decreasing order. The percentage variance in the data captured by each principal component is thereby calculated using the formula,

$$\%\mathrm{var}_{i}=\frac{\lambda_{i}}{\sum_{j=1}^{I} \lambda_{j}}$$

where, $\lambda_{i}$ is the $i^{th}$eigen value of the correlation matrix, $XX^{T}$. For a given data matrix, the total number of principal components equal the number of original variables. However, the principal components are arranged in decreasing order of the corresponding eigen values so that the first component describes the direction of maximum variation in the data and the next component describes the next maximum variation in the data perpendicular to the first and so on. Often, the first few components describe the most important variations while the last few components are attributed to noise in the data [1]. While PCA allows for identifying relationships between the TFs (or conditions), it can do so only with TFs and conditions in isolation. Additionally, techniques like biclustering allow the identification of relationships amongst the genes and conditions simultaneously. Extension to the PCA algorithm like the spectral biclustering which co-clusters gene and conditions during the SVD have been used to address this issue [2]. However, we chose Evolutionary Biclustering for our purpose due to its major advantages of efficient exploration of parameter space and better controls on the quality of the bicluster [3]

*PCA results identify major clusters of conditions and the corresponding markers*

From the representation in Figure S2 (b), the experimental conditions of different growth factors and their combinations appear to segregate in specific clusters which we designate as Groups A, B and C. Group A contains the conditions involving BMP4 along with either WNT3A or PI3KI or both, with BMP4 being the dominant factor in this combination. Upon overlaying the transcription factor expression data with growth factor combinations it appears that HNF4α, *HNF1β* and *PDX1*, which are markers for more mature pancreatic lineages, occur alongside Group A conditions. Thus, in our case BMP4 in presence of PI3K inhibition and Wnt signaling may be suitable for later stage maturation of the stem cells. Group B conditions contain FGF2 as a major factor along with WNT3A. It is found that both pluripotency (*OCT4*) and the endoderm factors (*CER* and *HNF6*) are relatively favored by conditions involving FGF2 as the major contributor. Many of the conditions in Group C involve PI3K inhibition and are associated with most of the important endoderm TFs. Activin+PI3K inhibition (point P in Figure 4) was found to work well for endoderm induction as seen in the study by McLean *et al*. [4]. Interestingly, FGF2 is found to favor DE in combination with BMP4 as seen in several recent studies [5, 6]. Thereby, PCA could identify many of the known facts about endoderm differentiation from our experimental data sets.

**Figure S2 (a) Percentage of the total variance in the data captured by the subsequent principal components on PCA analysis of the mean expression data.** The left Y axis represents the percentage variance of the original data explained by each PC while the right Y axis represents the cumulative variance explained by the first *n* components. The first three components explain about 68% of the variation in the data. **(b)** **Biplot of genes and conditions in the co-ordinate space of the first two PCs. (c) Scree plot showing the eigen values associated with each of the PCs.** The scree plot shows several elbows, after the 2^nd^, 4^th^ and the 7^th^ components. **(d) The major genes favored by the different conditions when considering the first three PCs.**

References

1. Abdi H, Williams LJ: **Principal component analysis.** *Wiley Interdisciplinary reviews: Computational Statistics* 2010, **2:**433-459.

2. Kluger Y, Basri R, Chang JT, Gerstein M: **Spectral biclustering of microarray data: coclustering genes and conditions.** *Genome research* 2003, **13:**703-716.

3. Divina F, Aguilar-Ruiz JS: **Biclustering of expression data with evolutionary computation.** *Knowledge and Data Engineering, IEEE Transactions on* 2006, **18:**590-602.

4. McLean AB, D'Amour KA, Jones KL, Krishnamoorthy M, Kulik MJ, Reynolds DM, Sheppard AM, Liu H, Xu Y, Baetge EE: **Activin a efficiently specifies definitive endoderm from human embryonic stem cells only when phosphatidylinositol 3 kinase signaling is suppressed.** *Stem Cells* 2007, **25:**29-38.

5. Xu X, Browning V, Odorico J: **Activin, BMP and FGF pathways cooperate to promote endoderm and pancreatic lineage cell differentiation from human embryonic stem cells.** *Mechanisms of Development* 2011.

6. Yu P, Pan G, Yu J, Thomson JA: **FGF2 Sustains NANOG and Switches the Outcome of BMP4-Induced Human Embryonic Stem Cell Differentiation.** *Cell stem cell* 2011, **8:**326-334.
