# Supplementary material for: Analysis of alternative signaling pathways of endoderm induction of human embryonic stem cells identifies context specific differences
Source: BMC Syst Biol. 2012 Dec 15;6:154. doi: 10.1186/1752-0509-6-154 (PMC3547704; doi:10.1186/1752-0509-6-154)
Supplement: Additional file 2 — Selection of biclustering parameters.docx [file 1752-0509-6-154-S2.docx]

*Effect of model parameters on Biclustering*

In a recent work by Zhang *et al.*, the SEBI algorithm was applied to a transcriptional factor data-set of embryonic stem cells [1]. The SEBI algorithm was successful in identifying biologically relevant biclusters stable under the free parameters of the algorithm. This section elaborates the selection of free parameters of the SEBI algorithm.

GA Parameters

GA has been shown to be efficient in solving this class of NP hard problems, but the common criticism in using GA is its lack of convergence criteria and sensitivity to various search parameters. In the present simulation, a population size of 20 was used which was simulated for 700 generations, at which point no further improvement of the optimal objective was observed. A crossover probability of 0.5 and a mutation probability of 0.2 were used to maintain sufficient diversity in the population. Table S3 summarizes all the GA parameters in detail.

Bicluster Parameters

While parameters associated with the GA formulation influences the optimal objective, there are additional parameters associated with the biclustering formulation which affects the quality of optimal bicluster. Equation 1 in the text details the objective function for optimizing the bicluster formulation, which consists of the following free parameters: $\delta$, the user defined threshold on residue; $W_{c}$, $W_{r}$ the relative weights associated with the columns and the rows of the bicluster respectively. The optimal bicluster obtained is significantly affected by the values of these parameters.

In order to analyze the effect of these parameters on the optimum bicluster, the optimization problem was solved at various values of *δ*, *W_c_* and *W_r_*, as summarized in FiguresS3 (a-e). Figure S (a) shows the variation of the number of genes and conditions in the optimal bicluster when the threshold on the residue in varied. Very low threshold identifies smaller biclusters. For example, low values of $\delta$ = 0.5 identifies optimal biclusters containing 2 genes and 2 conditions. Increasing the threshold relaxes the problem and therefore, the algorithm can search for biclusters with acceptable residue as well as larger volumes. The size of the biclusters increases with the relaxation of the threshold residue. Larger thresholds, however, compromise the quality of the bicluster, hence we select a value of $\delta$ = 1.5 which gives optimal biclusters containing 3 genes and 5 conditions and acceptable residue.

Figure S3 (b-e) illustrates the effect of the relative weights on columns and rows on the volume of the identified optimal bicluster and on the number of identified genes and conditions in the bicluster. The weights *W_c_* and *W_r_* allow user the flexibility to bias the bicluster to include more genes or more conditions. Such flexibility is useful with prior knowledge of the structure of existing network. Comparing Figures S3 (b), (d), it is found that the bicluster volume does not change appreciably with changes in row weight and the column weight, the volume increases from 10 to 30 when changing *W_r_* from 0.5 to 2 while it changes from 12 to 24 over the same range. For $W_{c}$ greater than 2, we see rapid increase in the number of conditions because the search is sensitive to $W_{c}$. Figure S3 (c), (e) further breaks up the volume into genes and conditions and illustrates how it changes in the number of both genes and conditions with $W_{c}$ and $W_{r}$ respectively. We find that increasing the column (row) weight increases the number of conditions (genes) while the number of genes (conditions) remain almost constant until $W_{c}$ (*W_r_*) = 2.

Figure S3 (f) shows the effect of row and column weights on the residue of the bicluster for a fixed threshold value of $\delta$ = 1.5. Changing the row weights is found to increase the residue appreciably. However, the residue is found to be less sensitive to the column weights. It is interesting to note that the residue is never found to be higher than the threshold even though this check was not explicitly introduced in the formulation.

Following above analysis, we chose the value of $\delta$ = 1.5 in order to capture reasonable volume of the bicluster. Regarding the weights *W_r_* and *W_c_* , in the absence of prior knowledge regarding the structure of expected bicluster, all were chosen to be on the lower end of 1.

*Effect of model parameters on the robust subsets*

While the bootstrap + biclustering algorithm enables determination of biclusters which remain robust to experimental noise, these are still evaluated for certain specific values of model parameters. Hence to analyze its sensitivity to the model parameters, the entire procedure was repeated for different values of model parameters: the threshold on residue (*δ*); row weight (*W_r_*) and column weights (*W_c_*). The frequency of occurrence of the two groups was subsequently measured by changing the parameter values, as illustrated in Figure S3 (g-i). Figure S3 (g) shows the variation in the frequency of occurrence of the robust bicluster for varying values of $\delta$. It was observed that for a broad range of the threshold the subsets are being repeated over 50% of time. Also, for low values of $\delta$, the number of repeats of Group 1 remains almost constant indicating that it is indeed robust. At larger values of $\delta$, the number of repeats for this group decrease and Group 2 takes over. Higher values of $\delta$ relax the constraint on the residue of the biclusters and therefore, increase the volume of the biclusters and the residue. Hence, this increases the occurrence of other genes and conditions in the biclusters and therefore, we see a decrease in the number of repeats for Group 1. It is interesting to note that the number of repeats for Group 2 increases with delta indicating that it is possibly the next robust subset present in the array but has higher residue as compared to Group 1. Figure S3 (h) shows the variation in the number of repeats with the column weight. Again, we see that the number of repeats for Group 1 goes through a maximum at 1.0 and on average stays above 500. At lower $W_{c}$, the biclusters are very small and therefore, the subsets are repeated fewer number of times and the repeats increase with $W_{c}$. However, when the $\delta$ crosses 1.0, the larger biclusters tend to have relatively high residue and thus contain mostly genes-condition groups with less similar profiles. Thus, we see a decrease in the repeats at larger $W_{c}$. Again, we note that Group 2 subset occurs more frequently with increase in $W_{c}$. Figure S3 (i) shows the variation in the number of repeats with the row weight.


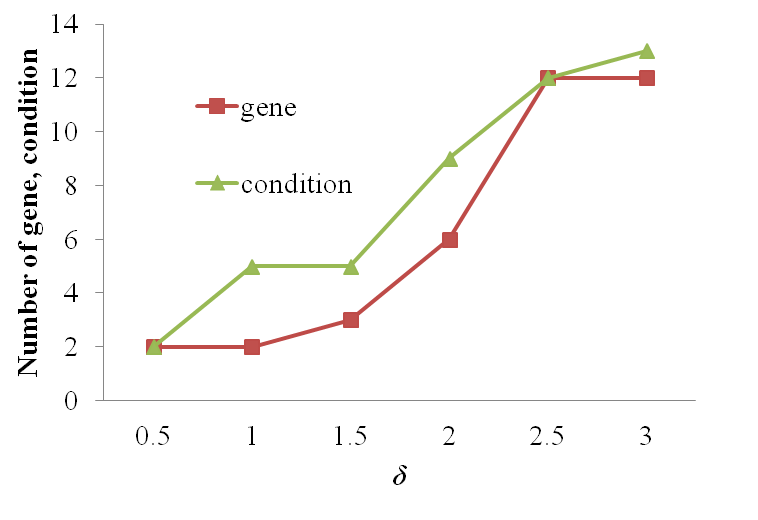


**Figure S3 (a): Variation of number of genes and conditions in the optimal bicluster with different values of the threshold,** ***δ.*** Increasing the threshold increases the number of genes and conditions contained by the optimal bicluster. A rapid increase in the number of TFs and conditions is observed after a *δ* of 1.5.


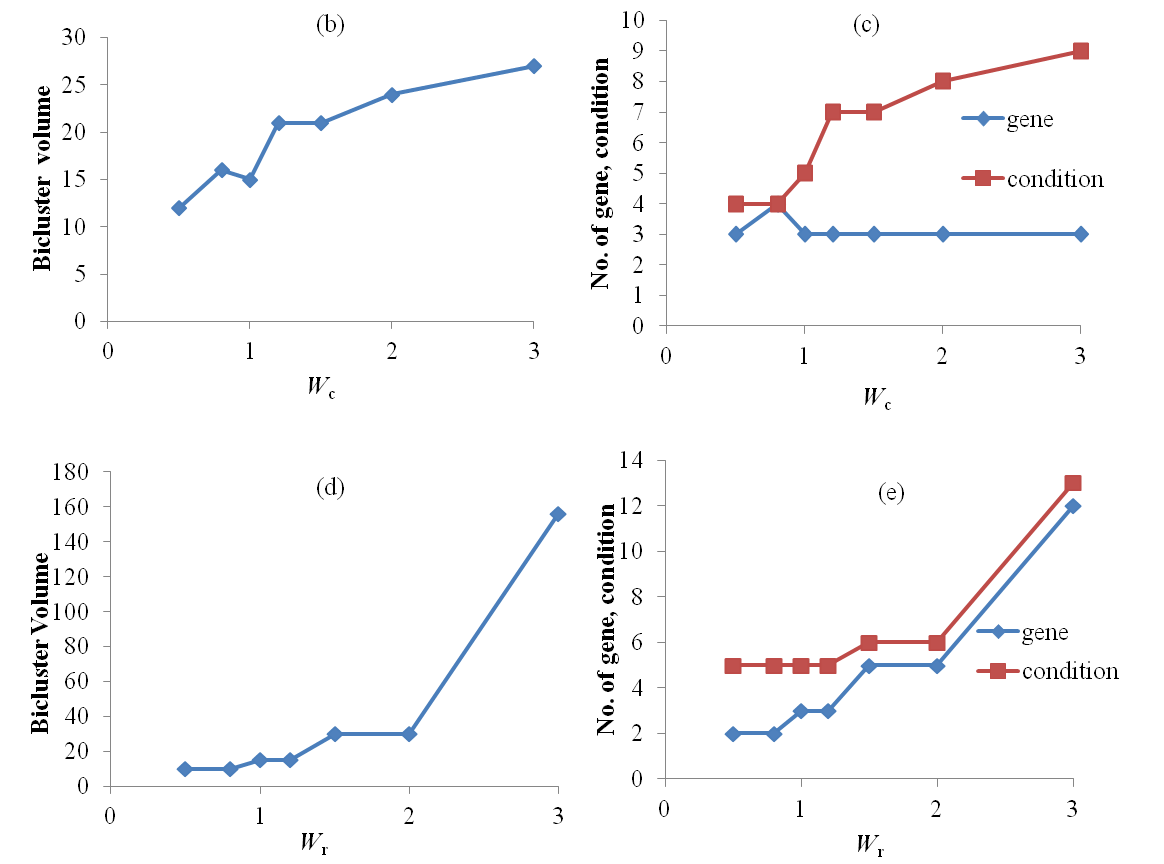


**Figure S3 (b-e): Effect of model parameters on features of optimal bicluster**. Variation in the bicluster volume and the number of genes, conditions in the optimal bicluster with changes in the column weights (b-c) and row weights (d-e) respectively.


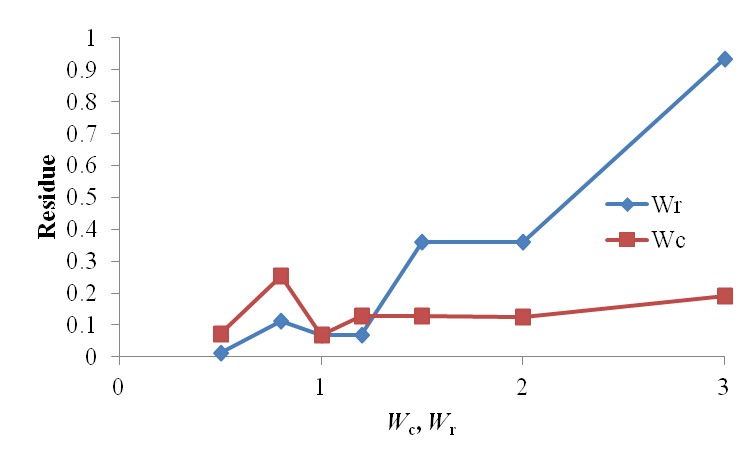


**Figure S3 (f): Variation of the residue as a function of row and column weights.** The residue is found to be sensitive to the row weights. All the residues remain well within the threshold limit of *δ* = 1.5.


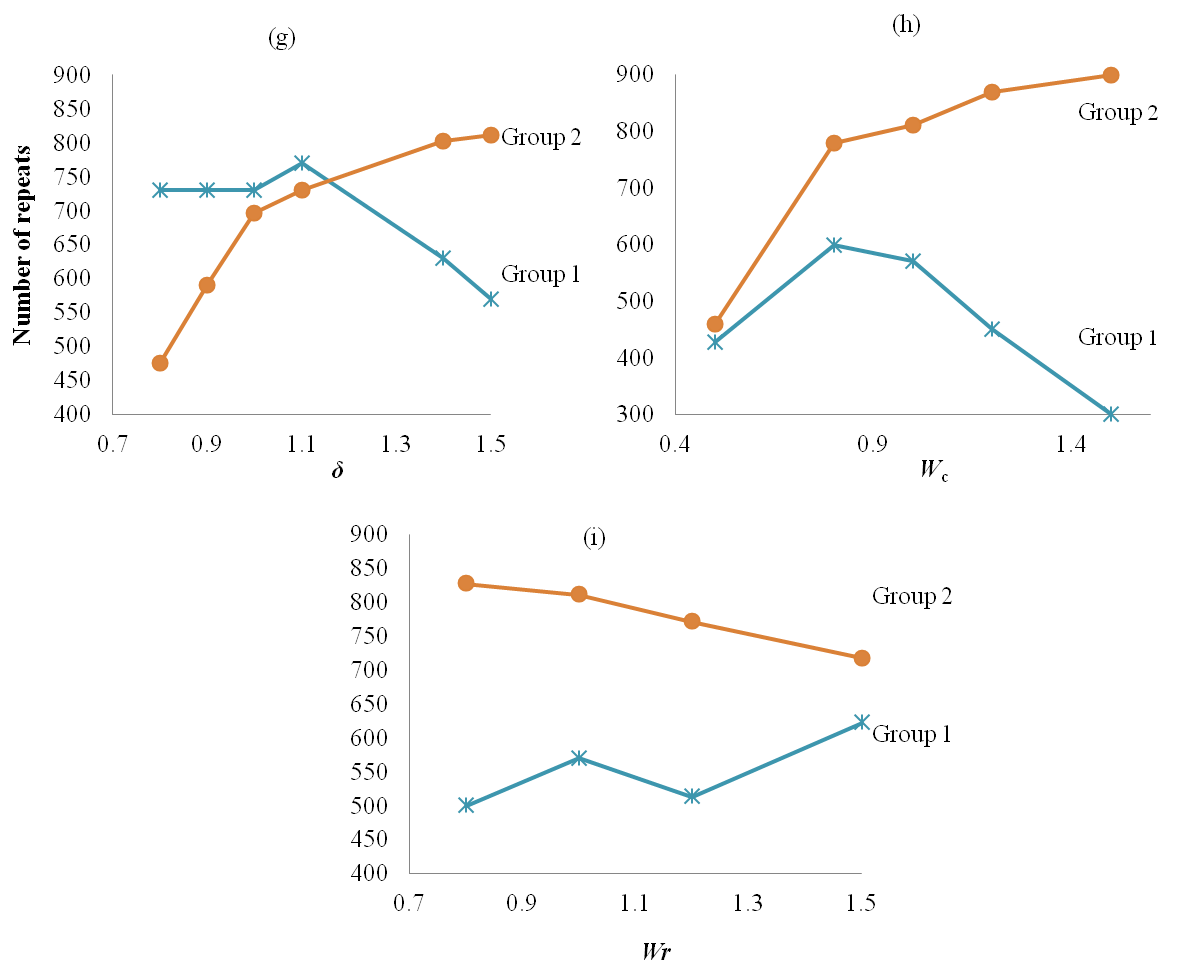


**Figure S3 (g-i): Sensitivity of the identified robust bi-cluster on model parameters**. Bi-clustering of the bootstrap data identifies 2 groups of robust bi-clusters. The figure illustrates the number of repeats of these robust bi-clusters with changes in (g) threshold, *δ* (h) column weight, *W*_c_. (i) row weight, *W*_r_

**Table S3: Summary of the GA parameters.**

| **Parameter** | **Value** |
| --- | --- |
| Population Size | 20 |
| Number of generations | 700 |
| Crossover probability | 0.5 |
| Mutation probability | 0.2 |
| Elitism probability | 1 |
| Weight for conditions ($W_{e}$) | 1 |
| Weight for genes ($W_{r}$) | 1 |

References

1. Zhang X, Jaramillo M, Singh S, Kumta P, Banerjee I: **Analysis of Regulatory Network Involved in Mechanical Induction of Embryonic Stem Cell Differentiation.** *PLoS One* 2012, **7:**e35700.
