# Supplementary material for: Analysis of alternative signaling pathways of endoderm induction of human embryonic stem cells identifies context specific differences
Source: BMC Syst Biol. 2012 Dec 15;6:154. doi: 10.1186/1752-0509-6-154 (PMC3547704; doi:10.1186/1752-0509-6-154)
Supplement: Additional file 3 — Transcription factors and primers list.docx [file 1752-0509-6-154-S3.docx]

Table S1. TFs selected to mark the stages of definitive endoderm induction along with their primers

| **MARKER (TFs)** | **PRIMERS FOR qRT-PCR** | **Reference** |
| --- | --- | --- |
| OCT4 | CTGGGTTGATCCTCGGACCT | (D'Amour, Bang et al. 2006) |
|  | CACAGAACTCATACGGCGGG |  |
| CXCR4 | CACCGCATCTGGAGAACCA | (D'Amour, Bang et al. 2006) |
|  | GCCCATTTCCTCGGTGTAGTT |  |
| SOX17 | CTCTGCCTCCTCCACGAA | (Osafune, Caron et al. 2008) |
|  | CAGAATCCAGACCTGCACAA |  |
| BRACHYURY | TGCTTCCCTGAGACCCAGTT | (D'Amour, Bang et al. 2006) |
|  | GATCACTTCTTTCCTTTGCATCAAG |  |
| PTF1α | GAAGGTCATCATCTGCCATCG | (D'Amour, Bang et al. 2006) |
|  | GGCCATAATCAGGGTCGCT |  |
| PDX1 | AAGTCTACCAAAGCTCACGCG | (Kroon, Martinson et al. 2008) |
|  | GTAGGCGCCGCCTGC |  |
| CER | ACAGTGCCCTTCAGCCAGACT | (D'Amour, Bang et al. 2006) |
|  | ACAACTACTTTTTCACAGCCTTCGT |  |
| FOXA2 (HNF3β) | GGAGCGGTGAAGATGGAA | (Osafune, Caron et al. 2008) |
|  | TACGTGTTCATGCCGTTCAT |  |
| GATA4 | GGAAGCCCAAGAACCTGAAT | (Rust, Sadasivam et al. 2006) |
|  | GGGAGGAAGGCTCTCACTG |  |
| HNF1β | TCACAGATACCAGCAGCATCAGT | (Kroon, Martinson et al. 2008) |
|  | GGGCATCACCAGGCTTGTA |  |
| HNF4α | CATGGCCAAGATTGACAACCT | (Kroon, Martinson et al. 2008) |
|  | TTCCCATATGTTCCTGCATCAG |  |
| HNF6 | TGTGGAAGTGGCTGCAGGA | (Zhang, Jiang et al. 2009) |
|  | TGTGAAGACCAACCTGGGCT |  |
| GAPDH | ACGACCACTTTGTCAAGCTCATTTC | (D'Amour, Bang et al. 2006) |
|  | GCAGTGAGGGTCTCTCTCTTCCTCT |  |
